# Re-Assessment of PrP^Sc^ Distribution in Sporadic and Variant CJD

**DOI:** 10.1371/journal.pone.0066352

**Published:** 2013-07-03

**Authors:** Richard Rubenstein, Binggong Chang

**Affiliations:** Departments of Neurology and Physiology/Pharmacology, State University of New York Downstate Medical Center, Brooklyn, New York, United States of America; New York University, United States of America

## Abstract

Human prion diseases are fatal neurodegenerative disorders associated with an accumulation of PrP^Sc^ in the central nervous system (CNS). Of the human prion diseases, sporadic Creutzfeldt-Jakob disease (sCJD), which has no known origin, is the most common form while variant CJD (vCJD) is an acquired human prion disease reported to differ from other human prion diseases in its neurological, neuropathological, and biochemical phenotype. Peripheral tissue involvement in prion disease, as judged by PrP^Sc^ accumulation in the tonsil, spleen, and lymph node has been reported in vCJD as well as several animal models of prion diseases. However, this distribution of PrP^Sc^ has not been consistently reported for sCJD. We reexamined CNS and non-CNS tissue distribution and levels of PrP^Sc^ in both sCJD and vCJD. Using a sensitive immunoassay, termed SOFIA, we also assessed PrP^Sc^ levels in human body fluids from sCJD as well as in vCJD-infected humanized transgenic mice (Tg666). Unexpectedly, the levels of PrP^Sc^ in non-CNS human tissues (spleens, lymph nodes, tonsils) from both sCJD and vCJD did not differ significantly and, as expected, were several logs lower than in the brain. Using protein misfolding cyclic amplification (PMCA) followed by SOFIA, PrP^Sc^ was detected in cerebrospinal fluid (CSF), but not in urine or blood, in sCJD patients. In addition, using PMCA and SOFIA, we demonstrated that blood from vCJD-infected Tg666 mice showing clinical disease contained prion disease-associated seeding activity although the data was not statistically significant likely due to the limited number of samples examined. These studies provide a comparison of PrP^Sc^ in sCJD vs. vCJD as well as analysis of body fluids. Further, these studies also provide circumstantial evidence that in human prion diseases, as in the animal prion diseases, a direct comparison and intraspecies correlation cannot be made between the levels of PrP^Sc^ and infectivity.

## Introduction

Prion diseases are a group of fatal neurodegenerative disorders that affect both animals and humans. Included are Creutzfeldt-Jakob disease (CJD) in humans, bovine spongiform encephalopathy (BSE, or “mad cow disease”) in cattle which was first reported in the United Kingdom in 1986, chronic wasting disease (CWD) in mule deer and elk, and scrapie in sheep. Of the various forms of CJD, sporadic CJD (sCJD) is the most common but has unknown etiology while the variant form (vCJD) is related to eating meat tainted with BSE. Human and animal prion diseases all have long presymptomatic incubation periods but are typically rapidly progressive once clinical symptoms begin. Prion diseases are associated with conversion of the native, α-helix-rich cellular prion protein (PrP^C^) into an aberrantly folded β-pleated-enriched isoform (PrP^Sc^) that has a tendency to aggregate and accumulate [1]. Although prion infectivity is most readily isolated from the CNS, PrP^C^ is widely distributed in extraneuronal tissues, especially in patients with vCJD [2]–[4]. In blood, for instance, PrP^C^ is mainly found in the soluble plasma fraction, but is also found on white blood cells (WBCs) as well as platelets [5]. The expression of PrP^C^ on follicular dendritic cells in secondary lymphoid organs is required for prion transmission through peripheral routes of inoculation [6]–[8]. Experimental inoculations and subsequent passages in animal models, such as mice, hamsters, sheep, elk, and macaques, have shown variable levels of prion infectivity in tissues, such as spleen and muscle, as well as in plasma and buffy coat WBC [9]. The level of infectivity in blood is estimated to be lower than 100 infectious units per ml in mice inoculated with mouse-adapted human infectious agent, orders of magnitude lower than that in the brain [10]–[11].

CJD is the predominant human prion disease. CJD can be divided into classical and variant forms. Classical CJD exists as either a familial (fCJD), sporadic (sCJD), or iatrogenic (iCJD) form. Variant Creutzfeldt-Jakob disease (vCJD) was first described in the United Kingdom in 1996 and has been linked to exposure to meat from BSE affected cattle.

Recently, several cases of prion transmission were reported in the UK. These patients contracted CJD years after having received non-leukodepleted blood products from asymptomatic donors who were later diagnosed with vCJD [12]–[14]. These incidences have ignited debate over the presence of prion infectivity in blood and highlighted the urgent need to develop an antemortem blood test to identify presymptomatic CJD cases and to safeguard the blood supply. Several biological markers have been proposed, but the presence of PrP^Sc^ remains the only reliable and specific indicator of prion diseases.

There is currently no single pre-mortem diagnostic test for classical or vCJD. A definitive confirmation of CJD diagnosis is by brain biopsy or autopsy for detection of: PrP^Sc^ by Western blotting, scrapie-associated fibrils by negative stain electron microscopy, or the presence of PrP^Sc^-containing amyloid plaques by immunohistochemistry. A diagnosis of probable, classical CJD can be made by observing EEG patterns (periodic sharp wave complexes), a positive 14-3-3 cerebrospinal fluid (CSF) assay, and high signal abnormalities in magnetic resonance imaging (MRI) of the caudate nucleus and/or putamen on diffusion-weighted imaging or fluid attenuated inversion recovery. A differential diagnosis of fCJD requires genetic testing for mutations in the PrP gene. In the case of vCJD, EEGs and CSF are not as useful as in classical CJD. A positive result using a CSF 14-3-3 protein assay and measurement of CSF tau protein may be indicative of patients with vCJD [15]–[16]. For vCJD, MRI scans have been informative as well as detection of PrP^Sc^ in tonsils. All of these tests have limited utility for confirmatory antemortem diagnosis and lack sufficient sensitivity and specificity. In the CSF 14-3-3, tau and S100β proteins are useful diagnostic markers of sCJD, although CSF tau showed better overall diagnostic accuracy than 14-3-3 or S100β [17].

The goal for CJD diagnosis continues to be using less invasive samples, such as body fluids, for the detection of PrP^Sc^, the most specific biomarker to date. Two recent studies [18]–[19] reported the use of the real-time quaking-induced conversion (RT-QuIC) assay with CSF samples for detection of CJD. Atarashi et al [18] reported greater than 83% sensitivity and 100% specificity compared to 72% sensitivity and 86% specificity when the samples were assayed for 14-3-3. Similarly, in a blinded study, McGuire et al [19] reported that CSF from sCJD patients analyzed for PrP^Sc^ by RT-QuIC had a sensitivity and specificity of 87% and 100%, respectively.

Surround optical fiber immunoassay (SOFIA) alone or, depending on the starting samples, in combination with target amplification by protein misfolding cyclic amplification (PMCA) [20] has been successfully used with tissues and body fluids for the preclinical detection and clinical diagnosis of sheep scrapie and CWD [21]–[22]. In this manuscript we describe the use of SOFIA for detection of PrP^Sc^ or prion disease associated seeding activity (PASA) in CSF from sCJD patients as well as a comparison of PrP^Sc^ in the central nervous system (CNS) and non-CNS tissues from sCJD and vCJD.

## Results

**Figure 1:**
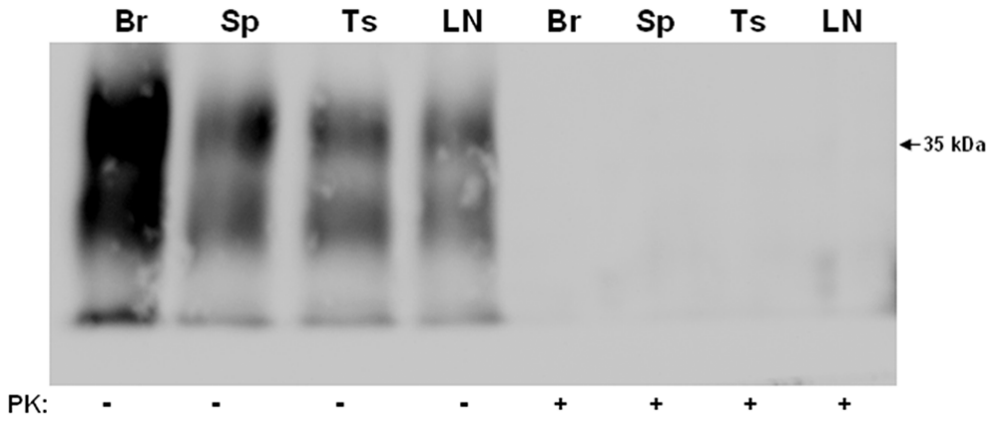
Western blot analysis of PrP^C^ from normal human tissues. Ten percent homogenates from normal human brain (Br), spleen (Sp), tonsil (Ts), and lymph node (LN) were prepared and ultracentrifuged through a sucrose cushion as described in Materials and Methods. Pellets were resuspended and either untreated or PK-treated prior to western blot analysis with Mab 3F4.

**Figure 2:**
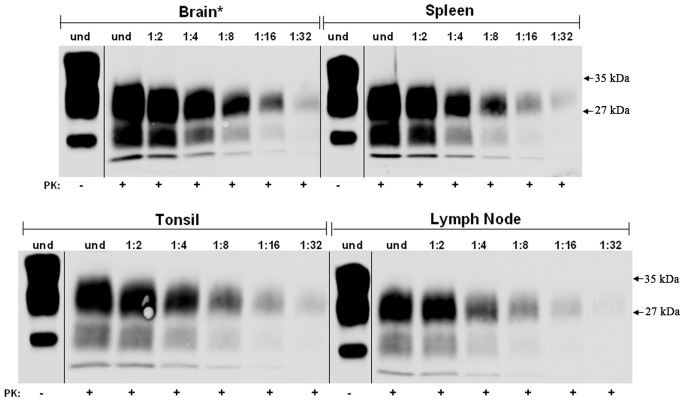
Partially purified, protease-resistant PrP^Sc^ titration from sCJD tissues. Homogenates of sCJD brain, spleen, tonsil, and lymph node were prepared, concentrated by ultracentrifugation and the pellets were resuspended as described in Materials and Methods. Undiluted (und) and two-fold serial dilutions of the PK-treated resuspended pellets were western blotted and immunostained for PrP^Sc^ using Mab 3F4. A PK-untreated sample is shown for each tissue to demonstrate the completeness of the PK treatment. Asterisk (*) denotes that the amount of brain tissue equivalents loaded in each lane is 1/100 the amount relative to spleen, tonsil, and lymph node.

**Figure 3:**
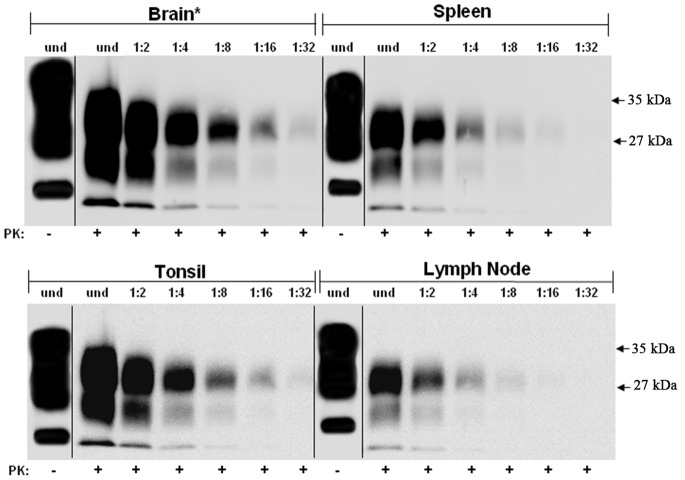
Partially purified, protease-resistant PrP^Sc^ titration from vCJD tissues. Homogenates of vCJD brain, spleen, tonsil, and lymph node were prepared and concentrated by ultracentrifugation as described in Materials and Methods. Undiluted (und) and two-fold serial dilutions of the PK-treated resuspended pellets were western blotted and immunostained for PrP^Sc^ using Mab 3F4. A PK-untreated sample is shown for each tissue to demonstrate the completeness of the PK treatment. Asterisk (*) denotes that the amount of brain tissue equivalents loaded in each lane is 1/100 the amount relative to spleen, tonsil, and lymph node.

PrP was concentrated by ultracentrifugation of 10% homogenates from brain, spleen, tonsil and lymph node from non-CJD individuals and the samples were analyzed by Western Blotting with or without PK-treatment using Mab 3F4 (Figure 1). The blots show, as expected, the 3 protein bands in the non-PK treated samples while no PrP^C^ was observed in the PK-treated samples. This demonstrated that the PK treatment used in this study is sufficient to completely degrade any PrP^C^ in these preparations. The same sample concentration protocol was used with brain, spleen, tonsil and lymph node samples from cases of sCJD and vCJD (Figures 2 and 3, respectively). The concentrated homogenates were untreated or PK-treated and then serial two-fold dilutions were analyzed on western blots. As noted in the Materials and Methods section, the “undiluted” (and subsequent serially diluted) spleen, tonsil, and lymph node samples for western blotting contained 100 times more tissue equivalents than the “undiluted” (and subsequent serially diluted) brain tissue. All sCJD tissues exhibited the same end-point dilution of 1∶32 (Figure 2). Based on previous reports this was unexpected since: (i) the CNS from prion disease infected tissues contain the highest levels of infectivity and, (ii) previous studies [23] have reported that PrP^Sc^ was present in vCJD spleens, tonsils, and lymph nodes, but absent in sCJD. In the vCJD samples used in this study, the results were similar to that for the sCJD samples. In the case of vCJD brain, after 100 times less tissue equivalents was applied relative to the non-CNS tissues, the vCJD brain and tonsil had end-point dilutions of 1∶32, while spleen and lymph node were only detectable to the 1∶16 dilutions (Figure 3). With the exception of brain, 10% homogenates of all other sCJD and vCJD tissues that were analyzed on western blots without concentration did not exhibit any PrP^Sc^ immunostaining (data not shown).

The degree of variability in the levels of PrP^Sc^ was assessed for all the samples within each tissue group as well as across tissue groups for both sCJD and vCJD using end-point dilution and/or densitometric analysis of the PrP^Sc^ bands (not shown). There were no statistically significant differences in the levels of PrP^Sc^ of all the samples within each tissue group for sCJD. That is, the levels of PrP^Sc^ from all the sCJD brain samples were similar to each other as was also the case for the sCJD spleen, tonsil and lymph node samples in each of those respective groups. Likewise the same statistically insignificant levels of variability in PrP^Sc^ quantitation were observed when comparing the samples within each of the vCJD tissue groups (brain, spleen, tonsil, lymph node). In the case of tonsil samples, an overall comparision of vCJD vs. sCJD suggests higher levels of PrP^Sc^ in the former but the differences did not reach the level of statistical significance.

**Figure 4:**
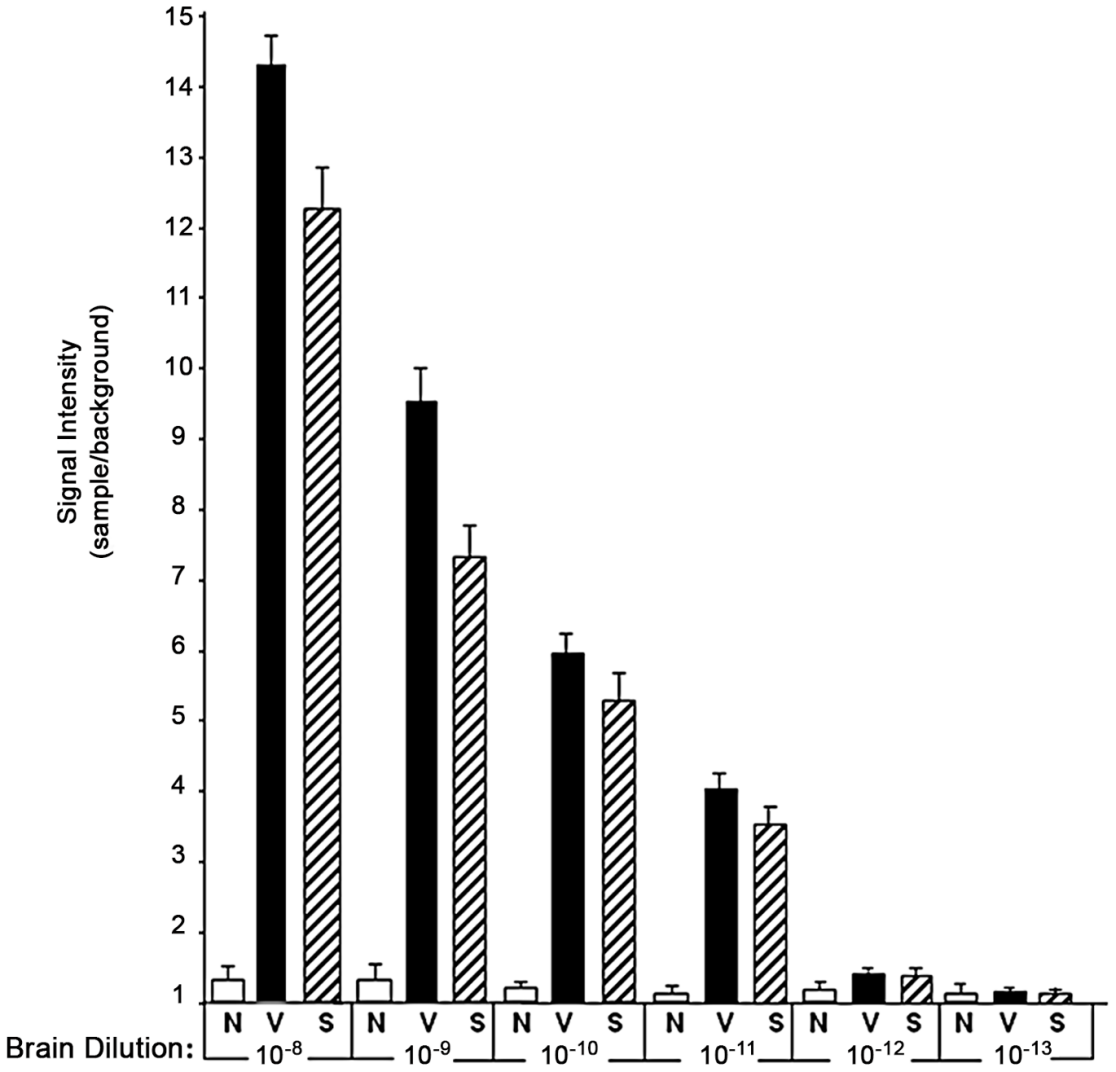
Effects of sCJD and vCJD brain dilution on PrP^Sc^ detection by SOFIA. Ten percent homogenates from all the normal human (N), vCJD (V), and sCJD brain tissues were prepared as described in Materials and Methods. Serial 10-fold dilutions were prepared from each brain tissue sample and analyzed in triplicate by SOFIA as described. For each sample, data was calculated as the mean fluorescent signal±standard deviation and expressed as the sample to background ratio (signal intensity).

We next determined the most suitable antibody combination to use for both capture ELISA and SOFIA. We tested over 50 combinations of anti-PrP monoclonal antibodies (Mabs) by capture ELISA using PK-untreated normal, sCJD and vCJD brain tissues. Several Mab combinations gave similar results where the signal intensity of 10^−3^ dilutions of normal brain tissue was similar to background while the sCJD and vCJD tissue readings were significantly higher (7X; data not shown). Based on these results, we chose the combination of Mab 3F4 for capture and biotinylated Mab 5D6 for detection of PrP. Serial ten-fold dilutions of sCJD and vCJD brain homogenates demonstrated that the capture ELISA signal intensities for the infected tissues continued to decrease and could no longer be distinguished from both the normal brain tissue and background readings at a 10^−5^ dilution. In addition, capture ELISA using 10% homogenates of spleen, tonsil, and lymph node from sCJD and vCJD yielded a signal 2X normal only if first concentrated by immunoprecipitation with no readings above normal tissues and/or background after a 10^−2^ dilution (data not shown). On the other hand, using SOFIA for PrP^Sc^ detection, values for unconcentrated CJD brain homogenates did not approach normal brain homogenate (and background) values until 10^−13^ dilution (Figure 4) indicating that SOFIA is 8 logs more sensitive than capture ELISA for sCJD and vCJD. In spleen, the end-point using SOFIA was a 10^−11^ dilution for both sCJD and vCJD, while for tonsil and lymph node the end-point for both was reached at a 10^−10^ dilution (Table 1).

**Table 1:** End-Point Titrations of PrP^Sc^ by SOFIA in non-CNS Tissues.

				SOFIA					
	Normal			sCJD			vCJD		
Dilution	Spleen	Tonsil	Lymph Node	Spleen	Tonsil	Lymph Node	Spleen	Tonsil	Lymph Node
10^−8^	1.3*	1.0	1.1	6.9	6.5	5.9	7.7	7.0	6.7
10^−9^	1.3	1.2	1.2	3.9	3.1	3.4	4.1	3.4	3.8
10^−10^	1.4	1.0	1.2	2.6	1.5	1.3	2.8	1.5	1.4
10^−11^	1.2	1.2	1.3	1.4	1.2	1.3	1.2	1.1	1.3
10^−12^	1.2	1.0	1.1	1.2	1.2	1.2	1.2	1.0	1.2

* Values are voltage readings expressed as the means of the sample background ratio.

**Figure 5:**
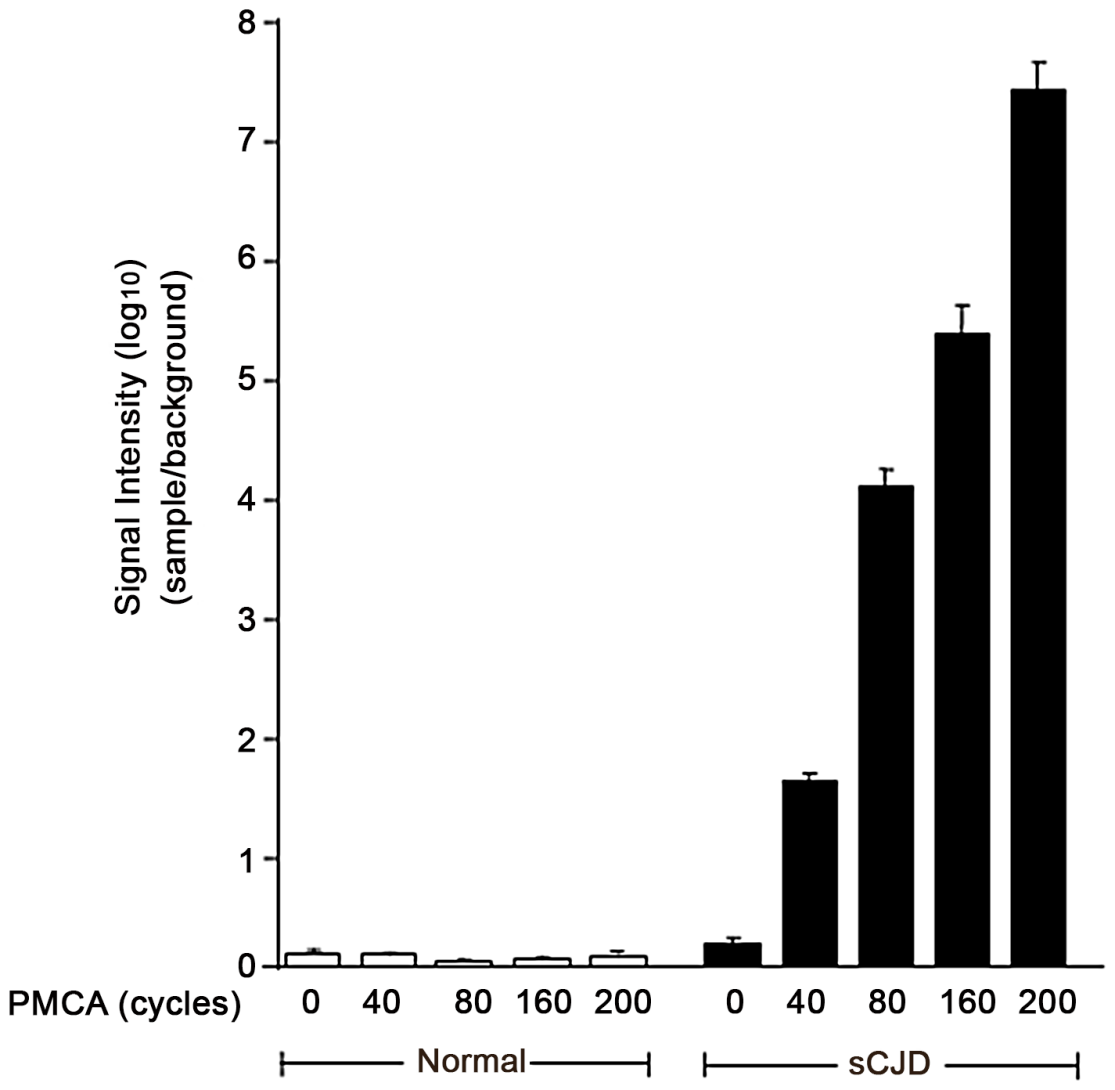
Detection of PrP^Sc^ from sCJD by SOFIA following PASA of human CSF. PASA was carried out using normal human and sCJD CSF as the source of the seeding material. Serial PMCA was carried out individually on all of the normal human and sCJD CSF for a maximum of 200 cycles with samples collected at 0, 40, 80, 160, and 200 cycles for IP and analysis in triplicate by SOFIA. For each sample, data was calculated as the mean fluorescent signal±standard deviation and expressed as the sample to background ratio (signal intensity). Signal intensities from SOFIA were adjusted based on the dilution of samples throughout sPMCA.

**Figure 6:**
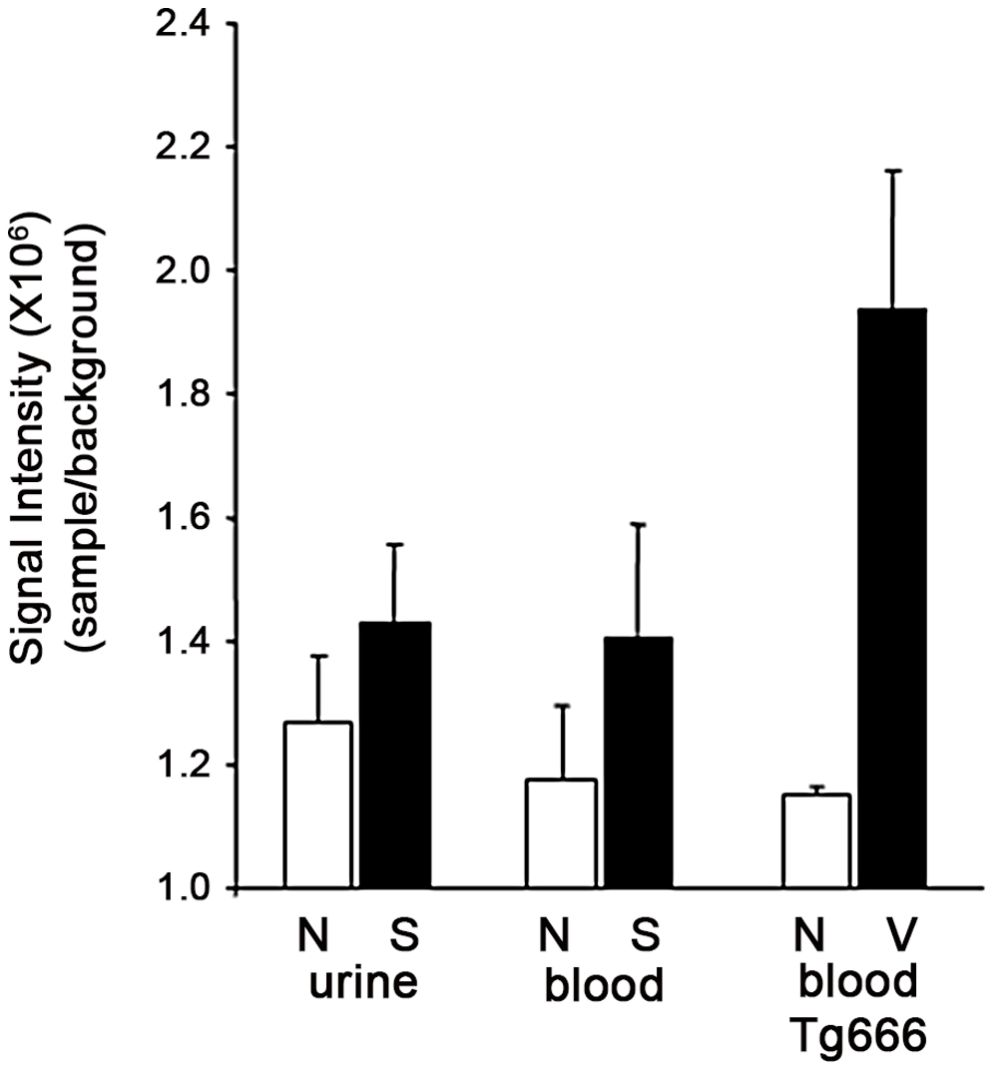
PrP^Sc^ Detection by SOFIA Following PASA (sPMCA_200_) in urine and blood. PASA was evaluated in human urine and blood from each sCJD case and blood from vCJD-infected Tg666 mice. Following sPMCA_200_, samples were analyzed in triplicate by SOFIA for the presence of PrP^Sc^. Signal intensities from SOFIA were adjusted based on the dilution of samples throughout sPMCA_200_, calculated as the mean fluorescent signal±standard deviation and expressed as the signal/background ratio.

**Figure 7:**
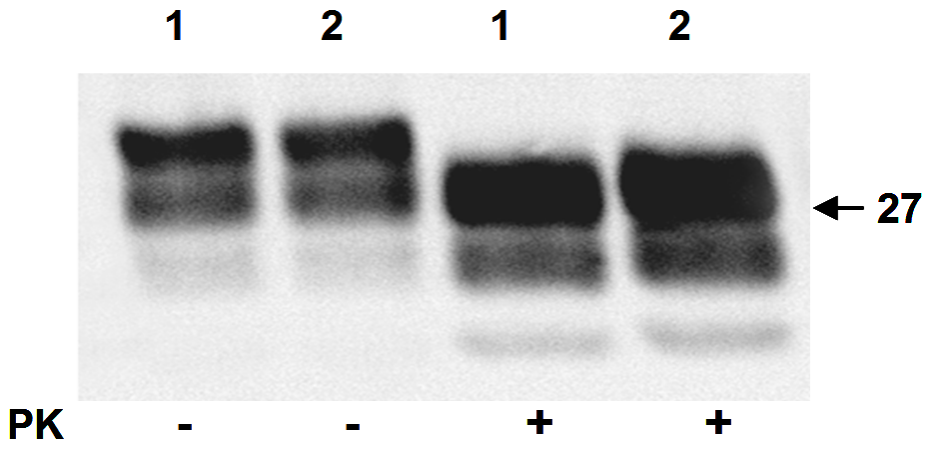
Western blot analysis of PrP^Sc^ from vCJD-infected Tg666 mice. Ten percent brain homogenates from two vCJD-infected Tg666 mice were prepared in lysis buffer and centrifuged at 13,000×g for 10 min. Twenty µl of supernatant from each of the two mice (1, 2) was either untreated or PK-treated prior to western blotting and immunostaining with Mab 3F4 as described in Materials and Methods.

We also analyzed the utility of SOFIA for detecting PASA in body fluids. Prior to sPMCA, PASA was undetectable by SOFIA in sCJD CSF (Figure 5). However, PASA could be demonstrated in CSF from sCJD after sPMCA_40–200_ by detection of the newly formed PrP^Sc^ using SOFIA (Figure 5). Based on SOFIA, all CSF samples from sCJD cases (but not the control CSF samples) demonstrated seeding activity for the PrP^C^ to PrP^Sc^ conversion with a rise in PrP^Sc^ levels as the number of sPMCA cycles increased from 40 to 200. Similar studies were performed with blood and urine samples from sCJD and normal controls. In contrast to the CSF data, neither blood nor urine from sCJD provided PASA for up to 200 cycles of sPMCA as demonstrated by similar background-like SOFIA results for the sCJD and control samples (Figure 6). Unfortunately, body fluids from vCJD patients were not available for comparison. However, as a surrogate source of vCJD blood, we utilized vCJD-infected Tg666 mice. Brain and blood from infected Tg666 mice, with incubation periods of 194 and 200 days, and normal Tg666 mice were analyzed. Western blotting of infected Tg666 brain homogenates demonstrated the presence of PK-resistant PrP^Sc^ confirming the disease in these animals (Figure 7). Blood samples from the vCJD-infected Tg666 were evaluated for PASA by performing sPMCA_200_ followed by SOFIA for detection of the amplified product (Figure 6). Although there is an obvious trend suggesting PASA in Tg666 blood following sPMCA_200_, comparison with normal Tg666 blood was not statistically significant (p = 0.12 for Mann-Whitney two-tailed test) possibly due to the small sample size.

## Discussion

The finding that CJD can be transmitted by transfusion of blood from pre-symptomatic vCJD victims placed some urgency on re-assessing the distribution of PrP^Sc^ in all forms of CJD. In addition, efforts in this direction offer the prospect of true antemortem diagnostic tests for these invariably fatal diseases. SOFIA alone or following PMCA has been used for the detection of PrP^Sc^ from tissues and body fluids of preclinical and symptomatic scrapie infected sheep and cervids with chronic wasting disease [21]–[22], [24]. Here we report the use of this technology for the detection of PASA in human prion diseases. Similar to our previous findings, detection of PASA by SOFIA in CNS tissues is more sensitive by orders of magnitude than other immunoassays. If we assume that a 10^−9^ dilution of sCJD brain contains 1–2 fg of PrP^Sc^ [18] our results indicate that SOFIA is capable of detecting on the order of 0.01–0.02 fg of PrP^Sc^. This is consistent with our previous studies of PASA detection using SOFIA with brain tissue from clinical animals infected with prion disease [25].

Furthermore, the sensitivity of SOFIA has allowed for detection of PrP^Sc^ in tissues of the lymphoreticular system, not only in vCJD samples, but also, for the first time, in sCJD tonsil and lymph node. Several studies [23], [26] reported that PrP^Sc^ is not detectable in the tonsils, spleen, or lymph nodes of patients with sCJD. However, a subsequent report [3] indicated that PrP^Sc^ could be detected in spleen and muscle of some sCJD patients on western blots if the PrP^Sc^ in the samples was first concentrated by sodium phosphotungstic acid precipitation to increase immunoassay sensitivity [2], [27]. In addition, Herzog et al [28] used sodium phosphotungstic acid precipitation followed by a sensitive ELISA on vCJD and sCJD infected primate tissues to show that while PrP^Sc^ levels were robust in vCJD-infected spleen, tonsil, and lymph node, only low levels of PrP^Sc^ were detected from sCJD spleen. In contrast to our findings, previous studies demonstrated that PrP^Sc^ was consistently less abundant in peripheral organs from sCJD tissues than in vCJD tissues. As noted in our studies, following sample concentration and immunoblotting of the non-CNS tissues, we did not find significant differences in PrP^Sc^ levels when comparing sCJD tissues between groups (spleens vs, lymph nodes vs. tonsils) or tissue samples within each group. In comparison to sCJD, spleens and lymph nodes from vCJD had less PrP^Sc^ while tonsils as a group appeared to be greater though not statistically significant. In the brain, the levels of PrP^Sc^ in both sCJD and vCJD were similar to each other and approximately 100 times greater than in the non-CNS tissues. This assumes that PrP^Sc^ from CNS and non-CNS tissues: (i) are structurally and physiochemically similar and thus precipitate equivalently under constant conditions, and (ii) are equally resistant to PK digestion. Although less than the amount detected in brain, the relatively higher-than-expected levels of PrP^Sc^ found in the non-CNS sCJD tissues is not concordant with the low levels of infectivity reported for spleen and lymph node [29]. However, as has been reported previously [24], [30], and demonstrated in this manuscript, the level of PrP^Sc^ does not necessarily parallel the level of infectivity for prion diseases.

CSF is produced by the choroid plexus, a network of capillaries found in the ventricular system of the brain, as an ultrafiltrate of plasma [31]. In healthy individuals, about 80% of the proteins found in CSF are derived from plasma and 20% from the brain. Although the ratio can be altered under pathological conditions, the bulk of the CSF proteins remain plasma-derived. With the concern about the possible transmission of prion disease by blood [32] as prion infectivity has been detected early in the animal models of disease in lymphoreticular tissues [33] the need for antemortem diagnosis and sensitive blood screening is clear. Previous studies have shown that RT-QUIC could be used for the detection of PASA in the CSF of prion diseased hamsters and sheep, as well as in sCJD and iCJD [18], [19], [34]–[35]. In this study we describe the use of SOFIA for the detection of PASA, a surrogate marker for infectivity, in CSF from sCJD patients. CSF from sCJD patients is a source of PASA for sPMCA and SOFIA was readily able to detect the amplified PrP^Sc^.

PASA was not detectable in sCJD blood or urine even after sPMCA_200._ It is possible, although unlikely, that increasing PMCA beyond 200 cycles might have generated detectable PASA although increasing the number of cycles also increases the risk of generating false positive signals [36]. Unequivocal experimental transmission of prion disease using blood from infected animals, but not from humans with sCJD, has been demonstrated. The ability to transmit prion diseases through blood transfusion was previously demonstrated in experimentally infected hamsters even during the asymptomatic phase of infection [37]–[38]. Successful transmission of disease after intracerebral injection of blood or plasma taken from sporadic or iatrogenic CJD patients [39]–[42] was not confirmed in other studies [43] and chimpanzees transfused with blood taken from sCJD patients failed to develop disease [43]–[44]. Whether infectivity is present in the blood of sCJD patients remains unsettled. Injection of blood or blood components from sCJD patients into experimental animals occasionally and inconsistently has resulted in transmission of the disease.

PrP^Sc^ has been detected in sheep, deer, and hamster blood, both at terminal stages of disease and in pre-symptomatic animals [21], [36], [45]–[48] and in CSF and urine from naturally and experimentally infected animals [22], [34], [48]–[50]. Furthermore, epidemiologic studies have demonstrated the presence of prion infectivity in blood and plasma from vCJD cases [51]–[54]. It is possible that the level of infectivity and/or PrP^Sc^ is too low to be detected or that there may be various forms of sCJD with subtle differences, which reflects varied amounts of infectivity in the blood.

We have shown that PrP^Sc^ is detectable by western blotting of samples from CNS and lymphoid tissues, including tonsil, of both vCJD and sCJD patients. We also demonstrated that PrP^Sc^ is detectable by SOFIA in unconcentrated, and even highly diluted, CNS and non-CNS tissues from human prion disease samples, as well as in CSF from sCJD patients. Thus, our assay could be used for antemortem diagnosis of both sCJD and vCJD. In addition, our data suggests that PMCA of blood followed by SOFIA should be pursued as a non-invasive assay for antemortem diagnosis of vCJD.

## Materials and Methods

### Ethics Statement

This study was carried out in strict accordance with the recommendations in the Guide for the Care and Use of Laboratory Animals of the National Institutes of Health. The protocol was approved by the Institutional Animal Care and Use Committee at SUNY Downstate Medical Center (07-468-10).

### Tissues and Body Fluids

Sporadic CJD and normal samples were obtained from the National Prion Disease Pathology Surveillance Center (Case Western Reserve University, Cleveland, OH). The National CJD Research and Surveillance Unit (Edinburgh) supplied normal, sCJD and vCJD samples. All samples were anonymized prior to being received. In total, the number of individual samples from different cases consisted of: (a) normal: brain (13), spleen (3), tonsil (3), lymph node (3), CSF (10), blood (10), urine (10), (b) sCJD: brain (13), spleen (3), tonsil (3), lymph node (3), CSF (10), blood (10), urine (10), and (c) vCJD: brain (8), spleen (4), tonsil (3), and lymph node (4). We were unable to obtain any body fluids from vCJD patients.

Surrogate blood samples were produced by infecting humanized transgenic mice (Tg666) [55] with vCJD samples. Tg666 mice were injected intracerebrally with 20 µl, or intraperitoneally with100 µl, of a 20% human vCJD brain homogenate prepared in phosphate buffered saline (PBS) and monitored for clinical disease. Mice showing signs of disease (somnolence, kyphosis, tremors, stilted gait, ataxia) for three consecutive weeks were sacrificed by cardiac exsanguination followed by removal and freezing of brains. Blood samples were centrifuged at 13,000×g and the supernatants collected and frozen.

### Sample Preparations

For direct analysis of tissues (brain, spleen, tonsil, lymph node) by SOFIA, a 10% homogenate (10^−1^) was prepared in lysis buffer [PBS containing 1% Nonidet P-40, 0.5% sodium deoxycholate, 5 mM EDTA]. The homogenates were clarified by centrifugation at 2,000×g for 2 min and serial 10-fold dilutions of the supernatants were prepared in PBS.

For sample concentration by ultracentrifugation, 20% tissue homogenates were prepared in lysis buffer followed by the addition of an equal volume of 20% sarkosyl in TBS (50 mM Tris HCl, pH 7.4, 150 mM NaCl). A 500 µl aliquot of each sample was mixed with 3 ml of TBS with 10% NaCl +0.1% sulfobetain (SB) 3–14 and layered over a 1.5 ml 20% sucrose cushion in TBS +10% NaCl +0.1% SB 3–14. Samples were ultracentrifuged (Beckman Optima, MLS-50 rotor, 127,000×g) for 1 hr at 4°C. The pellets were resuspended in 100 µl of TBS +0.1% SB 3–14. The equivalent of 0.5 µl of the resuspended pellet from brain homogenate and 50 µl of the resuspended pellets from spleen, tonsil, and lymph node were considered as “undiluted” samples for western blotting.

### Western Blot

For proteinase K (PK) treatment, PK (100 µg/ml final concentration) was added for 30 min at 50°C followed by the addition of 1% protease inhibitor cocktail. For western blotting, Laemmli sample buffer was added to a 1× concentration (60 mM Tris-HCl, pH 6.8, 0.1% SDS, 100 mM DTT, 5% glycerol, and 0.01% bromophenol blue, pH 6.8) and samples were heated at 100°C for 10 min, microcentrifuged at 400×g for 2 min and electrophoresed (12% acrylamide gels) at 150 V. After electrotransfer, the nitrocellulose membranes were blocked for 1 hr in 5% non-fat dry milk +0.1% Tween 20, washed three times in PBS +0.5% Tween 20 (PBST) and incubated with Mab 3F4 (2 µg/ml) for 1 hr. After three PBST washes, the membranes were incubated for 1 hr in goat anti-mouse lgG (Fab fragment) conjugated to horseradish peroxidase (1∶5000) (ThermoFisher). The membranes were washed in PBST and protein bands were detected using ECL supersignal west dura kit (ThermoFisher). All tissue samples were analyzed in either duplicate or triplicate depending on the amount of tissue obtained. Quantification of PrP^Sc^ band intensity was performed by densitometric analysis using NIH provided Image J software.

### PASA and SOFIA

For all body fluids (CSF, blood and urine), PASA was assessed on each sample by serial PMCA (sPMCA) followed by SOFIA using protocols similar to those previously described (21, 22). A single cycle of sPMCA consisted of combining 100 µl of each sample (CSF, blood or urine) with 50 µl of 10% normal human brain homogenate (NHBH) [prepared in phosphate-buffered saline (PBS) containing 1% Triton-X100, 4 mM EDTA, and 1% protease inhibitor cocktail (Calbiochem)] followed by sonication (QSONICA: 490W power setting, 39,000 J, amplitude of 60, 90 sec process time consisting of 3 sec on: 1 sec off) and incubation at 37°C for 1 hr. A second cycle began with the addition of another 50 µl aliquot of NHBH followed by sonication and incubation. After every 10 cycles, 500 µl of the sample was transferred to a new tube and PMCA continued. For our studies, we performed 40–200 cycles of serial PMCA (sPMCA_40_ - sPMCA_200_).

PMCA of the body fluids was followed by immunoprecipitation (IP). After sPMCA of 40 to 200 cycles (sPMCA_40_–_200_), a 100 µl sample was added to 50 µl of MagnaBind protein G - monoclonal antibody (Mab) 8B13 complex [prepared by mixing anti-PrP Mab 8B13 (10 mg/ml) with MagnaBind protein G (Pierce) at a 1∶10 ratio, incubating for 1 hr at room temperature followed by 3 washes in PBS] in a final volume of 1 ml PBS. The tubes were incubated overnight at 4°C with constant mixing. The beads were then washed 3 times with PBS, resuspended in 500 µl PBS, and heated at 100°C for 10 min. The supernatants collected from a 10 min centrifugation at 16,000×g were analyzed by SOFIA.

Each sample was analyzed in triplicate by SOFIA as previously described (21, 22). Mab 3F4 (5 µg/ml) was used as the capture reagent on 96-well high binding microtiter plates (Costar). After an overnight incubation, blocking buffer (3% bovine serum albumin/PBS) was added for 1 hr. Wells were washed followed by the addition of samples to be tested and incubation for 2 hrs. After additional washes, biotinylated Mab 5D6 (4 µg/ml) was added to each well for 1 hr followed by four washes with PBST and then a 1 hr incubation with streptavidin-rhodamine red-X conjugate (Invitrogen). Finally, 1 N NaOH was added followed by heating at 100°C for 15 min and neutralization with 1M Tris-HCl, pH 7.4. Samples were analyzed using SOFIA for detection of rhodamine red fluorescence.

### Statistics

Statistical significance of the densitometric quantitation of western blotted PrP^Sc^ was determined using the Student’s t-test. Mean values calculated from SOFIA among the normal and the infected groups were compared using the Mann-Whitney test and a p value <0.05 was defined as being statistically significant.

## References

[1] Pan KM, Baldwin M, Nguyen J, Gasset M, Serban A, et al (1993) Conversion of a-helices into bsheets features in the formation of the scrapie prion proteins. Proc Natl Acad Sci USA 90: 10962–10966. 7902575 10.1073/pnas.90.23.10962 PMC47901

[2] Wadsworth JDF, Joiner S, Hill AF, Campbell TA, Desbruslais M, et al (2001) Tissue distribution of protease resistant prion protein in variant Creutzfeldt–Jakob disease using a highly sensitive immunoblotting assay. Lancet 358: 171–180. 11476832 10.1016/s0140-6736(01)05403-4

[3] Glatzel M, Abela E, Maissen M, Aguzzi A (2003) Extraneural pathologic prion protein in sporadic Creutzfeldt-Jakob disease. N Engl J Med 349: 1812–1820. 14602879 10.1056/NEJMoa030351

[4] Ramasamy I, Law M, Collins S, Brook F (2003) Organ distribution of prion proteins in variant Creutzfeldt–Jakob disease. Lancet Infect Dis 3: 214–222. 12679264 10.1016/s1473-3099(03)00578-4

[5] Cashman NR, Loertscher R, Nalbantoglu J, Shaw I, Kascsak RJ, et al (1990) Cellular isoform of the scrapie agent protein participates in lymphocyte activation. Cell 61: 185–192. 1969332 10.1016/0092-8674(90)90225-4

[6] Fraser H, Brown KL, Stewart K, McConnell I, McBride P, et al (1996) Replication of scrapie in spleens of SCID mice follows reconstitution with wild-type mouse bone marrow. J Gen Virol 77: 1935–1940. 8760445 10.1099/0022-1317-77-8-1935

[7] Blättler T, Brandner S, Raeber AJ, Klein MA, Voigtländer T, et al (1997) PrP-expressing tissue required for transfer of scrapie infectivity from spleen to brain. Nature 389: 69–73. 9288968 10.1038/37981

[8] Brown KL, Stewart K, Ritchie DL, Mabbott NA, Williams A, et al (1999a) Scrapie replication in lymphoid tissues depends on prion protein-expressing follicular dendritic cells. Nat Med 5: 1308–1312. 10545999 10.1038/15264

[9] Brown P (2005) Blood infectivity, processing and screening tests in transmissible spongiform encephalopathy. Vox Sang 89: 63–70. 16101685 10.1111/j.1423-0410.2005.00683.x

[10] Brown P, Cervenakova L, McShane LM, Barber P, Rubenstein R, et al (1999b) Further studies of blood infectivity in an experimental model of transmissible spongiform encephalopathy, with an explanation of why blood components do not transmit Creutzfeldt–Jakob disease in humans. Transfusion 39: 1169–1178. 10604242 10.1046/j.1537-2995.1999.39111169.x

[11] Cervenakova L, Yakovleva O, McKenzie C, Kolchinsky S, McShane L, et al (2003) Similar levels of infectivity in the blood of mice infected with human-derived vCJD and GSS strains of transmissible spongiform encephalopathy. Transfusion 43: 1687–1694. 14641865 10.1046/j.0041-1132.2003.00586.x

[12] Llewelyn CA, Hewitt PE, Knight RS, Amar CAK, Cousens S, et al (2004) Possible transmission of variant Creutzfeldt–Jakob disease by blood transfusion. Lancet 363: 417–421. 14962520 10.1016/S0140-6736(04)15486-X

[13] Peden AH, Head MW, Ritchie DL, Bell JE, Ironside JW (2004) Preclinical vCJD after blood transfusion in a PRNP codon 129 heterozygous patient. Lancet 364: 527–529. 15302196 10.1016/S0140-6736(04)16811-6

[14] Wroe SJ, Pal S, Siddique D, Hyare H, Macfarlane R, et al (2006) Clinical presentation and pre-mortem diagnosis of variant Creutzfeldt–Jakob disease associated with blood transfusion: a case report. Lancet 368: 2061–2067. 17161728 10.1016/S0140-6736(06)69835-8

[15] Green AJ, Thompson EJ, Stewart GE, Zeidler M, McKenzie JM, et al (2001) Use of 14-3-3 and other brain-specific proteins in CSF in the diagnosis of variant Creutzfeldt-Jakob disease. J Neurol Neurosurg Psychiatry 70: 744. 11385008 10.1136/jnnp.70.6.744 PMC1737395

[16] Goodall CA, Head MW, Everington D, Ironside JW, Knight RS, et al (2006) Raised CSF phospho-tau concentrations in variant Creutzfeldt-Jakob disease: diagnostic and pathological implications. J Neurol Neurosurg Psychiatry 77: 89. 16361602 10.1136/jnnp.2005.065755 PMC2117383

[17] Coulthart MB, Jansen GH, Olsen E, Godal DL, Connolly T, et al (2011) Diagnostic accuracy of cerebrospinal fluid protein markers for sporadic Creutzfeldt-Jakob disease in Canada: a 6-year prospective study. BMC Neurology 11: 133. 22032272 10.1186/1471-2377-11-133 PMC3216246

[18] Atarashi R, Satoh K, Sano K, Fuse T, Yamaguchi N, et al (2011) Ultrasensitive human prion detection in cerebrospinal fluid by real-time quaking-induced conversion. Nat Med 17: 175–178. 21278748 10.1038/nm.2294

[19] McGuire LI, Peden AH, Orr CD, Wilham JM, Appleford NE (2012) Real Time Quaking-Induced Conversion Analysis of Cerebrospinal Fluid in Sporadic Creutzfeldt–Jakob Disease. Ann Neurol 72: 278–285. 22926858 10.1002/ana.23589 PMC3458796

[20] Saborio GP, Permanne B, Soto C (2001) Sensitive detection of pathological prion protein by cyclic amplification of protein misfolding. Nature 411: 810–813. 11459061 10.1038/35081095

[21] Rubenstein R, Chang B, Gray P, Piltch M, Bulgin MS, et al (2010) A novel method for preclinical detection of PrPSc in blood. J Gen Virol 91: 1883–1892. 20357038 10.1099/vir.0.020164-0

[22] Rubenstein R, Chang B, Gray P, Piltch M, Bulgin MS, et al (2011) Prion Disease Detection, PMCA Kinetics, and IgG in Urine from Sheep Naturally/Experimentally Infected with Scrapie and Deer with Preclinical/Clinical Chronic Wasting Disease. J Virol 85: 9031–9038. 21715495 10.1128/JVI.05111-11 PMC3165845

[23] Hill AF, Butterworth RJ, Joiner S, Jackson G, Rossor MN, et al (1999) Investigation of variant Creutzfeldt-Jakob disease and other human prion diseases with tonsil biopsy samples. Lancet 353: 183–189. 9923873 10.1016/s0140-6736(98)12075-5

[24] Rubenstein R, Bulgin MS, Chang B, Sorensen-Melson S, Petersen RB, et al (2012) PrPSc detection and infectivity in semen from scrapie-infected sheep. J Gen Virol 93: 1375–1383. 22323531 10.1099/vir.0.038802-0

[25] Chang B, Gray P, Piltch M, Bulgin MS, Sorensen-Melson S, et al (2009) Surround optical fiber immunoassay (SOFIA): An ultra-sensitive assay for prion protein detection. J Virol Meth 159: 15–22. 10.1016/j.jviromet.2009.02.019 19442839

[26] Head MW, Ritchie D, Smith N, McLoughlin V, Nailon W, et al (2004) Peripheral Tissue Involvement in Sporadic, Iatrogenic, and Variant Creutzfeldt-Jakob Disease An Immunohistochemical, Quantitative, and Biochemical Study. Am J Path 164: 143–153. 14695328 10.1016/S0002-9440(10)63105-7 PMC1602214

[27] Safar J, Wille H, Itri V, Groth D, Serban H, et al (1998) Eight prion strains have PrP(Sc) molecules with different conformations. Nat Med 4: 1157–1165. 9771749 10.1038/2654

[28] Herzog C, Riviere J, Lescoutra-Etchegaray N, Charbonnier A, Leblanc V, et al (2005) PrPTSE Distribution in a Primate Model of Variant, Sporadic, and Iatrogenic Creutzfeldt-Jakob Disease. J Virol 79: 14339–14345. 16254368 10.1128/JVI.79.22.14339-14345.2005 PMC1280201

[29] World Health Organization (2006) Guidelines on Tissue Infectivity Distribution in Transmissible Spongiform Encephalopathies. Geneva, Sept. 14–15, 2005.

[30] Miyazawa K, Emmerling K, Manuelidis L (2011) High CJD infectivity remains after prion protein is destroyed. J Cell Biochem 112: 3630–3637. 21793041 10.1002/jcb.23286 PMC3202053

[31] Thompson EJ (1995) Cerebrospinal fluid. J Neurol Neurosurg Psychiatry 59: 349–357. 7561910 10.1136/jnnp.59.4.349 PMC486067

[32] Aguzzi A (2000) Diseases, blood and the immune system: concerns and reality. Haematologica 85: 3–10. 10627667

[33] Mabbott NA, Farquhar CF, Brown KL, Bruce ME (1998) Involvement of the immune system in TSE pathogenesis. Immunol Today 19: 201–203. 9613034 10.1016/s0167-5699(98)01253-5

[34] Atarashi R, Wilham JM, Christensen L, Hughson AG, Moore RA, et al (2008) Simlified ultrasensitive prion detection by recombinant PrP conversion with shaking. 2008. Nat Methods 5: 211–212. 18309304 10.1038/nmeth0308-211

[35] Orrú CD, Wilham JM, Hughson AG, Raymond LD, McNally KL, et al (2009) Human variant Creutzfeldt–Jakob disease and sheep scrapie PrP^res^ detection using seeded conversion of recombinant prion protein. Protein Eng Des Sel 22: 515–521. 19570812 10.1093/protein/gzp031 PMC2719501

[36] Thorne L, Terry LA (2008) In vitro amplification of PrPSc derived from the brain and blood of sheep infected with scrapie. J Gen Virol 89: 3177–3184. 19008409 10.1099/vir.0.2008/004226-0

[37] Diringer H (1984) Sustained viremia in experimental hamster scrapie. Arch Virol 82: 105–109. 6437378 10.1007/BF01309373

[38] Casaccia P, Ladogana A, Xi YG, Pocchiari M (1989) Levels of infectivity in the blood throughout the incubation period of hamsters peripherally injected with scrapie. Arch Virol 108: 145–149. 2512893 10.1007/BF01313752

[39] Manuelidis EE, Kim JH, Mericangas JR, Manuelidis L (1985) Transmission to animals of Creutzfeldt-Jakob disease from human blood. Lancet 2: 896–897. 10.1016/s0140-6736(85)90165-5 2864612

[40] Tateishi J (1985) Transmission of Creutzfeldt-Jakob disease from human blood and urine into mice. Lancet 2: 1074. 10.1016/s0140-6736(85)90949-3 2865558

[41] Tamai Y, Kojima H, Kitajima R, Taguchi F, Ohtani Y, et al (1992) Demonstration of the transmissible agent in tissue from a pregnant woman with Creutzfeldt-Jakob disease. N Engl J Med 327: 649. 10.1056/NEJM199208273270918 1640969

[42] Deslys JP, Lasmézas C, Dormont D (1994) Selection of specific strains in iatrogenic Creutzfeldt-Jakob disease. Lancet 343: 848–849. 10.1016/s0140-6736(94)92046-x 7908088

[43] Brown P, Cervenáková L, Diringer H (2001) Blood infectivity and the prospects for a diagnostic screening test in Creutzfeldt-Jakob disease. J Lab Clin Med 2001 137: 5–13. 10.1067/mlc.2001.111951 11150018

[44] Brown P, Gibbs CJ Jr, Rodgers-Johnson P, Asher DM, Sulima MP, et al (1994) Human spongiform encephalopathy: the National Institutes of Health series of 300 cases of experimentally transmitted disease. Ann Neurol 35: 513–529. 8179297 10.1002/ana.410350504

[45] Castilla J, Saa P, Hetz C, Soto C (2005a) In vitro generation of infectious scrapie prions. Cell 121: 195–206. 15851027 10.1016/j.cell.2005.02.011

[46] Castilla J, Saa P, Soto C (2005b) Detection of prions in blood. Nat Med 11: 982–985. 16127436 10.1038/nm1286

[47] Saa P, Castilla J, Soto C (2006) Presymptomatic detection of prions in blood. Science 313: 92–94. 16825570 10.1126/science.1129051

[48] Murayama Y, Yoshioka M, Okada H, Takata M, Yokoyama T, et al (2007) Urinary excretion and blood level of prions in scrapie-infected hamsters. J Gen Virol 88: 2890–2898. 17872544 10.1099/vir.0.82786-0

[49] Gonzalez-Romero D, Barria MA, Leon P, Morales R, Soto C (2008) Detection of infectious prions in urine. FEBS Lett 582: 3161–3166. 18706416 10.1016/j.febslet.2008.08.003 PMC2593137

[50] Haley NJ, Seeli DM, Zabel MD, Telling GC, Hoover EA (2009) Detection of CWD prions in urine and saliva of deer by transgenic mouse bioassay. PLoS One 4: e4848. 19293928 10.1371/journal.pone.0004848 PMC2654070

[51] Hewitt PE, Llewelynm CA, MacKenziem J, Will RG (2006) Creutzfeldt- Jakob disease and blood transfusion: results of the UK Transfusion Medicine Epidemiological Review study. Vox Sang 91: 221–230. 16958834 10.1111/j.1423-0410.2006.00833.x

[52] Coste J, Prowse C, Eglin R, Fang C (2009) A report on transmissible spongiform encephalopathies and transfusion safety. Vox Sang 96: 284–291. 19220827 10.1111/j.1423-0410.2009.01161.x

[53] Chohan G, Llewelyn C, Mackenzie J, Cousens S, Kennedy A, et al (2010) Variant Creutzfeldt-Jakob disease in a transfusion recipient: coincidence or cause? Transfusion 50: 1003–1006. 20230536 10.1111/j.1537-2995.2010.02614.x

[54] Peden A, McCardle L, Head MW, Love S, Ward HJ, et al (2010) Variant CJD infection in the spleen of a neurologically asymptomatic UK adult patient with haemophilia. Haemophilia 16: 296–304. 20070383 10.1111/j.1365-2516.2009.02181.x

[55] Race B, Meade-White KD, Miller MW, Barbian KD, Rubenstein R, et al (2009) Susceptibilities of Nonhuman Primates to Chronic Wasting Disease. Emerg Infect Dis 15: 1366–1376. 19788803 10.3201/eid1509.090253 PMC2819871

